# Resource utilisation, costs and clinical outcomes in non-institutionalised patients with Alzheimer’s disease: 18-month UK results from the GERAS observational study

**DOI:** 10.1186/s12877-016-0371-6

**Published:** 2016-11-25

**Authors:** Alan Lenox-Smith, Catherine Reed, Jeremie Lebrec, Mark Belger, Roy W. Jones

**Affiliations:** 1 Eli Lilly and Company, Basingstoke, UK; 2 Eli Lilly and Company, Erl Wood, Windlesham UK; 3 Eli Lilly and Company, Bad Homburg, Germany; 4 RICE (The Research Institute for the Care of Older People), Royal United Hospital, Bath, UK

**Keywords:** Alzheimer’s disease, Clinical outcomes, Societal costs, AD dementia severity, Resource use, MMSE

## Abstract

**Background:**

Alzheimer’s disease (AD), the commonest cause of dementia, represents a significant cost to UK society. This analysis describes resource utilisation, costs and clinical outcomes in non-institutionalised patients with AD in the UK.

**Methods:**

The GERAS prospective observational study assessed societal costs associated with AD for patients and caregivers over 18 months, stratified according to baseline disease severity (mild, moderate, or moderately severe/severe [MS/S]). All patients enrolled had an informal caregiver willing to participate in the study. Healthcare resource utilisation was measured using the Resource Utilization in Dementia instrument, and 18-month costs estimated by applying unit costs of services and products (2010 values). Total societal costs were calculated using an opportunity cost approach.

**Results:**

Overall, 526 patients (200 mild, 180 moderate and 146 MS/S at baseline) were recruited from 24 UK centres. Mini-Mental State Examination (MMSE) scores deteriorated most markedly in the MS/S patient group, with declines of 3.6 points in the mild group, 3.5 points in the moderate group and 4.7 points in the MS/S group; between-group differences did not reach statistical significance. Patients with MS/S AD dementia at baseline were more likely to be institutionalised (Kaplan–Meier probability 28% versus 9% in patients with mild AD dementia; *p* < 0.001 for difference across all severities) and had a greater probability of death (Kaplan–Meier probability 15% versus 5%; *p* = 0.013) at 18 months. Greater disease severity at baseline was also associated with concomitant increases in caregiver time and mean total societal costs. Total societal costs of £43,560 over 18 months were estimated for the MS/S group, versus £25,865 for the mild group and £30,905 for the moderate group (*p* < 0.001). Of these costs, over 50% were related to informal caregiver costs at each AD dementia severity level.

**Conclusions:**

This study demonstrated a mean deterioration in MMSE score over 18 months in patients with AD. It also showed that AD is a costly disease, with costs increasing with disease severity, even when managed in the community: informal caregiver costs represented the main contributor to societal costs.

## Background

Alzheimer’s disease (AD) is the commonest cause of dementia representing 60–80% of all cases of dementia within Europe [1]. In the UK the Alzheimer’s Society has estimated that AD represents up to 62% of all cases of dementia [2]. Based on 2013 UK population data, the Alzheimer’s Society estimated the total age-standardised population prevalence of dementia to be 7% in those aged 65 years and over, equivalent to one in every 14 people in that age group [2]. At this rate of prevalence, approximately 850,000 individuals within the UK were expected to be affected by dementia in 2015 [2].

There have been several studies that have looked at the costs of dementia, most of which have used different methodology. Overall, and not surprisingly, there is general agreement that as the disease progresses the costs increase, especially once institutionalisation is required [3, 4]. Furthermore, informal care costs often form the majority of total costs [5]. As many of these and other studies use different methodologies ranging from multi-country clinical trial data and retrospective questionnaires, the robustness of the data has been questioned [6] and the difficulties of comparing studies has been highlighted [7].

Looking specifically at the UK, a recent analysis based on a model using estimates of prevalence, percentage of home versus care home usage, expenditure and quality of life, has estimated that the total cost of dementia to UK society is £26.3 billion per annum, representing an average annual cost of £32,250 per person [2]. Of this, approximately £4.3 billion, or 16%, is spent on healthcare costs, £10.3 billion (39%) on publicly and privately funded social care, and £0.1 billion (<1%) on other costs (including police time, research, advocacy and support by the voluntary sector). The remaining £11.6 billion (44%) represents the contribution of those unpaid family and friends who become carers for people with dementia [2]. The Alzheimer’s Society estimates that 670,000 people within the UK act as the primary, unpaid carer for an individual with dementia [8].

As the aforementioned studies lack robust, prospectively collected, local data, we performed an analysis of real-world UK data from the GERAS study. GERAS was a European prospective observational study that assessed the outcomes and societal costs associated with AD dementia for community-dwelling patients and their caregivers over at least 18 months. Patients were stratified according to their dementia severity at baseline into mild, moderate and moderately severe/severe disease groups, which broadly reflect the guidelines from NICE. The study design and baseline societal costs have been previously published [9]. The objective of the current paper is to describe the clinical outcomes in the UK in terms of cognition, institutionalisation and death, and to describe the costs seen in the different severity groups over an 18-month time period. The value of this study is its robustness, based on the prospective design, to gather national data on resource use and costs looking at patients with mild, moderate and severe AD dementia and the impact of this on their carers.

## Methods

The GERAS study was an 18-month, multicentre, prospective, non-interventional, observational cohort study conducted in three European countries: the UK, France and Germany [8]. The study was designed to reflect routine care in patients with AD dementia. All treatment decisions were at the discretion of the treating physician, and were made following standard care procedures during the course of normal clinical practice; all treatments were permissible.

The focus of the current analysis is on those data collected from the UK. Inclusion criteria were community-dwelling patients, aged at least 55 years, presenting within the normal course of clinical care, diagnosed with probable AD dementia according to the National Institute of Neurological and Communicative Disorders and Stroke and the Alzheimer’s Disease and Related Disorders Association criteria [10], with a Mini-Mental State Examination (MMSE) score [11] of ≤26 points, and with an informal caregiver who was willing to participate in the study and undertake responsibility for the patient for at least 6 months of the year. Enrolled patients were stratified according to baseline disease severity as having mild (MMSE 21–26 points), moderate (MMSE 15–20 points) or moderately severe/severe AD dementia (MMSE ≤14 points). Ethical review board approval of the study was obtained according to UK regulations and written informed consent was obtained from all participants or their caregivers.

Sample size for the GERAS study was calculated based on the number of patients required to determine total costs associated with resource use in each country with sufficient precision, assuming 30% of patients would be lost during 18 months of follow-up and an equal enrolment of patients into the three AD dementia severity groups. Based on these assumptions, a minimum of 200 patients per AD dementia severity group per country was required for a 95% confidence interval ±10% of mean cost at 18 months (total 600 patients from the UK).

Data were collected for patients and caregivers at baseline, and at 6, 12 and 18 months during routine care visits. Collected data included demographics, current medications, patient and caregiver health/social care resource use (using the Resource Utilization in Dementia [RUD] instrument) [12], and MMSE score [11]. Other outcomes data collected in the GERAS study (at baseline and 18 months only, not reported in this manuscript) include the cognitive subscale of the Alzheimer’s Disease Assessment Scale [13], patient functioning and behaviour (Alzheimer’s Disease Co-operative Study Activities of Daily Living Inventory [14, 15]), health-related quality of life (for both patients and caregivers, using the EuroQoL-5D instrument [16]), caregiver working status and caregiver burden (Zarit Burden Interview [17]). The analysis reported here focuses on specific clinical outcomes (change in cognition [MMSE score], institutionalisation and death) and costs over the 18 months of the study.

### Resource utilisation

The RUD instrument was used to measure healthcare resource utilisation by both patients and caregivers, and time spent on informal care by caregivers [12]. The RUD instrument, version RUD Complete 3.1, was administered by the physician, and answered by the primary informal caregiver, and the patient with AD when dementia severity permitted.

Resource use items collected included healthcare (outpatient visits [including: general practitioner, geriatrician, psychiatrist, neurologist, physiotherapist, occupational therapist, social worker and psychologist visits], hospitalisations, emergency room visits); community care services (district nurse, home aid, food delivery, day care, transportation, other); changes to patient living accommodation (permanent, temporary, institutionalisation); caregiver working status (working for pay, missing work days); and primary caregiver time spent caring for the patient. Caregiver time was calculated as the number of hours spent:

-  assisting the patient with basic activities of daily living (ADL) such as eating, bathing and dressing;
-  assisting with instrumental ADL such as housework and shopping; and
-  supervising the patient as required to prevent dangerous events such as risk of fire, and walking into the road if alone.

Information relating to patient medications, out-of-pocket expenses and additional financial support received, such as whether the patient or caregiver receives money from the government or an insurance company, was also collected during the interview with the caregiver. Data were collected from patients and caregivers at the baseline visit, and then during routine care visits at 6, 12 and 18 months (+/− 6 weeks [9]).

### Cost estimation

Eighteen-month costs were estimated by applying unit costs of services and products (2010 values) to the health and social care resource use collected over the 18-month follow-up period. For resource-use items, full details of the unit costs applied and their sources have already been reported (see the Supplementary Material in Wimo et al. 2013 [9]).

Total societal costs were calculated using an opportunity cost approach taking into account productivity loss for working caregivers and lost leisure time for non-working caregivers. Costs were calculated for the month before each visit. Total societal costs were calculated by adding patient healthcare costs (including medications, hospitalisations and outpatient visits), patient social care costs (including community care services, structural adaptations to the home and extra financial support) and caregiver informal care costs (including time spent giving care and missing work). The number of hours for basic and instrumental ADL were used to calculate caregiver time, with 24 hours per day availability for total caregiver time assumed, but time spent on supervision applied a zero cost value. The unit cost of caregiver time for working caregivers was considered to be the value of lost production time based on the national average wage per UK population (a unit cost of £15.65 per hour at 2010 values; see the Supplementary Material in Wimo et al. 2013 [9]); for non-working caregivers, the unit cost of caregiver time was considered to be the value of lost leisure time based on 35% of the national average wage per UK population. Mean costs are reported for each baseline severity group, with bootstrapped 95% confidence intervals of the means.

The following imputation rules were applied for missing data: for institutionalised patients, mean monthly costs from the last visit were used for the period until institutionalisation, then monthly costs for institutionalisation (standard monthly cost of care with zero other costs) were used from institutionalisation up to 18 months; for patients who died, last observation carried forward was used, such that costs from the last known visit were extrapolated up to the date of death (no costs after death were computed). For those patients with other reasons for discontinuation, multiple imputation regression method [18] stratified by MMSE group was performed on missing costs. The list of factors used in the multiple imputation procedure was selected from those identified by Dodel et al. [19]. In addition to the above-described base case approach, three sensitivity analyses of costs were performed applying alternative imputation rules for missing data.

### Statistical analyses

Demographics and baseline characteristics were summarised using descriptive statistics and were based on non-missing observations. In common with other clinical studies in AD [20–23], the change in MMSE score from baseline to 18 months was analysed using a mixed-model repeated measures analysis; this approach provides robust inference even in the presence of missing data. The model for the fixed effects included terms for AD dementia severity at baseline (classified as mild, moderate or moderately severe/severe), visit, and AD dementia severity at baseline-by-visit interaction. As the changes in MMSE score (rather than absolute scores) were being analysed, the inclusion of baseline AD dementia severity level as a covariate negated the need for including baseline demographics in the model.

The Kaplan–Meier method was used to describe the probabilities of institutionalisation and death, and differences across baseline AD dementia severity groups for these time-to-event endpoints were tested using the log-rank test. Descriptive statistics were used to describe baseline variables and discontinuations.

Comparisons across baseline AD dementia severity groups were made using analysis of variance (ANOVA) for continuous variables or the Cochran–Mantel–Haenszel test for categorical variables.

All analyses were performed using SAS version 9.2 (SAS Institute, Cary, NC, USA).

## Results

**Fig. 1:**
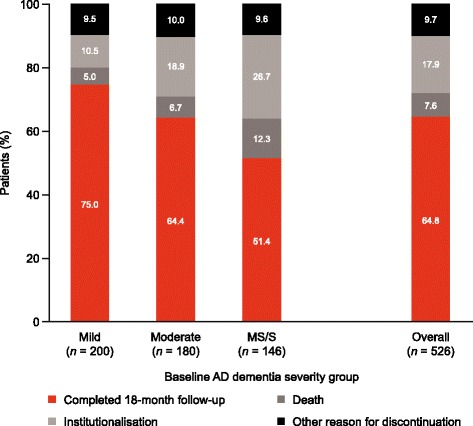
Patient disposition at 18 months according to baseline AD dementia severity group *AD* Alzheimer’s disease, *MS/S* moderately severe/severe

A total of 526 patients (200 mild, 180 moderate and 146 moderately severe/severe AD dementia) were recruited from 24 centres across the UK; baseline patient characteristics are shown in Table 1. Patient numbers at 18 months, when stratified according to baseline severity, are shown in Fig. 1. By the 18-month visit, 185 patients (35.2%) had discontinued the study due to institutionalisation (*n* = 94; 50.8% of discontinuations), death (*n* = 40; 21.6%) or other reasons (*n* = 51; 27.6%) e.g., lost to follow up, patient decision. Proportions of patients discontinuing increased with increasing baseline severity of AD dementia (Fig. 1).

**Table 1:** Patient demographics at baseline for the overall population stratified according to baseline AD dementia severity

Characteristic	Mild AD dementia(*N* = 200)	Moderate AD dementia (*N* = 180)	MS/S AD dementia (*N* = 146)	Overall (*N* = 526)	*P*-value^a^
Mean age in years [SD]	78.9 [6.7]	78.8 [8.1]	77.6 [8.7]	78.5 [7.8]	0.238
Gender, *n* female [%]	100 [50.0]	94 [52.2]	91 [62.3]	285 [54.2]	0.061
MMSE score, mean [SD]	23.1 [1.6]	17.8 [1.7]	8.7 [4.5]	17.3 [6.4]	N/A
Mean time since diagnosis in years [SD]	1.8 [2.1]	2.0 [1.9]	3.1 [2.2]	2.2 [2.1]	<0.001
Caregiver lives with patient, n [%]	156 [78.0]	129 [71.7]	122 [83.6]	407 [77.4]	0.037
Caregiver is spouse, *n* [%]	144 [72.0]	113 [62.8]	98 [67.1]	355 [67.5]	0.043^b^
Caregiver mean age in years [SD]	69.4 [11.70]	66.9 [11.77]	68.7 [12.21]	68.3 [11.90]	0.099
Caregiver gender, *n* female [%]	129 [64.5]	120 [66.7]	76 [52.1]	325 [61.8]	0.016
AD medication use, *n* [%]					0.012
No AD medication	31 [15.5]	29 [16.1]	19 [13.1]	79 [15.0]	−
Acetylcholinesterase inhibitor monotherapy	167 [83.5]	144 [80.0]	113 [77.9]	424 [80.8]	−
Memantine monotherapy	1 [0.5]	5 [2.8]	5 [3.4]	11 [2.1]	−
Acetylcholinesterase inhibitor + memantine	1 [0.5]	2 [1.1]	8 [5.5]	11 [2.1]	−

Percentages are based on the number of respondents (<1.0% missing)

*AD* Alzheimer’s disease, *ANOVA* analysis of variance, *MMSE* Mini-Mental State Examination, *MS/S* moderately severe/severe, *N/A* not applicable, *SD* standard deviation

^a^ANOVA *p-*value for continuous variables, Cochran–Mantel–Haenszel *p-*value for categorical variables between AD dementia severity groups

^b^ *P-*value relates to the relationship of the caregiver to the patient

### Clinical outcomes (MMSE)

#### Mean MMSE scores

**Fig. 2:**
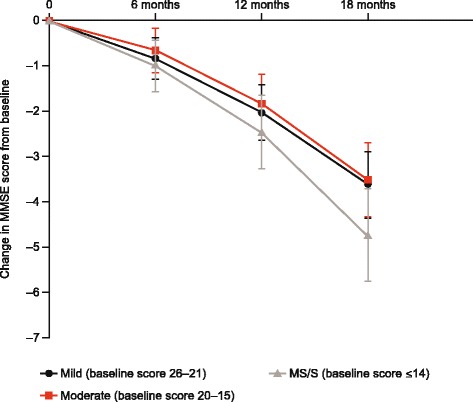
Change in MMSE score from baseline to 18 months stratified according to baseline AD dementia severity. *AD*, Alzheimer’s disease, *MMSE* Mini-Mental State Examination. Data are presented as least square means with 95% confidence intervals from mixed-model repeated measures analysis adjusted for baseline MMSE score. Change-from-baseline data missing for 0–9.0, 1–11.0 and 3.0–12.0% of patients across AD dementia severity groups at 6, 12 and 18 months, respectively

Over the 18-month duration of the GERAS study, a statistically significant deterioration in mean MMSE scores was observed (*p* < 0.001), which was most marked in the moderately severe/severe patient group (Fig. 2); declines in MMSE score were 3.6 points in the mild group, 3.5 points in the moderate group and 4.7 points in the moderately severe/severe group. However, differences in MMSE score changes between the groups did not reach statistical significance (*p* = 0.24).

### Severity level transitions

**Fig. 3:**
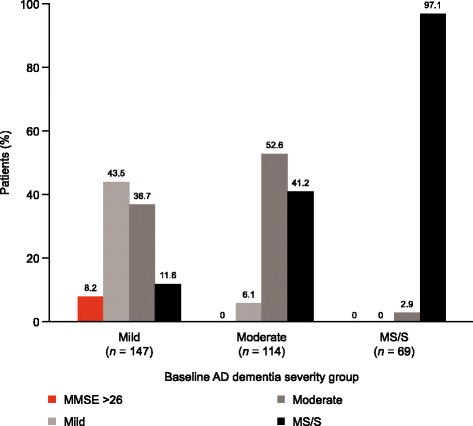
AD dementia status at 18 months stratified according to baseline severity. *AD*, Alzheimer’s disease, *MMSE* Mini-Mental State Examination. Percentages and patient numbers (*n*) shown exclude deaths and institutionalised patients

A total of 330 patients had a MMSE score at 18 months (64.8%). Among patients assessed as having mild disease at baseline, 43.5% remained with mild disease, 36.7% transitioned from mild to moderate disease, 11.6% transitioned from mild to moderately severe/severe disease, and 8.2% had an MMSE score of >26. For those with moderate disease at baseline, 6.1% had scores indicating mild disease, 52.6% still had moderate disease and 41.2% progressed to moderately severe/severe disease. Among patients with moderately severe/severe disease at baseline, 2.9% had MMSE scores for moderate disease, but the majority (97.1%) remained with moderately severe/severe disease (Fig. 3).

### Probability of institutionalisation

There was a significant difference in the probability of institutionalisation between the three AD dementia severity groups (*p* < 0.001 for overall difference between severities). Patients with moderately severe/severe AD dementia at baseline were more likely to be institutionalised at 18 months (Kaplan–Meier probability estimate, 28.0%) than those with mild AD dementia (9.0%) at baseline (Table 2). Overall, the probability of being institutionalised by 18 months was 17.2%.

**Table 2:** Probability of institutionalisation, or death, during the 18-month GERAS study, according to baseline AD dementia severity

	Mild AD dementia	Moderate AD dementia	MS/S AD dementia	Overall
Probability of being institutionalised at 18 months, %	9.0	17.9	28.0	17.2
95% CI	5.6; 14.3	12.7; 24.7	21.0; 36.7	14.1; 21.0
Probability of death at 18 months, %	5.3	6.5	14.9	8.2
95% CI	2.9; 9.6	3.5; 11.7	9.6; 22.7	6.0; 11.1

The Kaplan–Meier method was used to calculate probabilities and their associated 95% CIs

*AD* Alzheimer’s disease, *CI* confidence interval, *MS/S* moderately severe/severe

### Probability of death

There were also significant differences between the three severity groups in terms of probability of death (*p* = 0.013). At 18 months, the probability of death in those with moderately severe/severe AD dementia at baseline was greater than in those with mild AD dementia (14.9% compared with 5.3%). The probability of death at 18 months overall was 8.2% (Table 2).

### Caregiver time

**Fig. 4:**
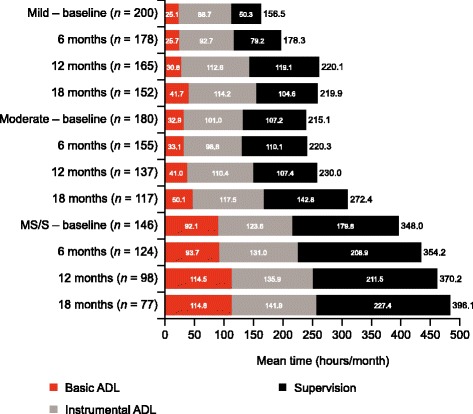
Caregiver time for activities of daily living stratified by baseline AD dementia severity. *AD* Alzheimer’s disease, *ADL* activities of daily living, *MS/S* moderately severe/severe. All values are based on data provided for the last month before each visit. The value beside each bar is the mean overall monthly caregiver time; this is not the sum of the three components of caregiver time because caregiver time was capped at 720 h/month. The *n* value is the number of respondents (0–9.0% missing). SD ranges observed across each individual endpoint at all time points and AD dementia severities were as follows: Overall time, 192–263 h; Supervision time, 120–269 h; Basic ADL, 59–138 h; Instrumental ADL, 83–150 h

At baseline, AD dementia severity was associated with increased mean total caregiver time (*p <* 0.001) and with increases in any of its components: time spent on basic or instrumental ADL or supervision (Fig. 4). Over the 18 months of the GERAS study, caregiver time spent on basic and instrumental ADL, together with time required for supervision, increased on average in all severity groups (Fig. 4). This increase from baseline was not significantly different across baseline AD dementia severity groups for any component of caregiver time or for overall caregiver time at 18 months.

### Societal costs

**Fig. 5:**
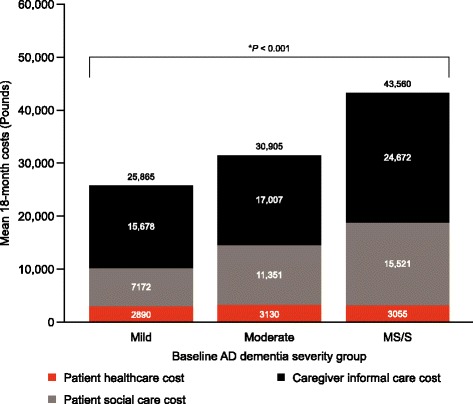
Estimated mean total societal costs of AD stratified according to baseline AD dementia severity, per patient. *AD* Alzheimer’s disease, *ANOVA* analysis of variance, *MS/S* moderately severe/severe. An opportunity cost approach was used for working and non-working caregivers; supervision time was excluded from caregiver time. Missing data were imputed at both the total societal cost and cost item level. The value above each column gives the mean total 18-month overall societal costs. This value is not the sum of the individual components as total societal costs were imputed separately from the imputation method used on the three cost components. *ANOVA *p*-value for comparison between AD dementia severity groups for total societal costs

The estimated mean total societal costs per patient over the 18-month duration of the study increased according to AD dementia severity, with total costs (bootstrapped 95% confidence intervals) of £43,560 (£39,059–£48,481) in patients with moderately severe/severe AD dementia compared with £25,865 (£23,444–£28,538) in those with mild and £30,905 (£28,539–£33,371) in those with moderate AD dementia (*p* < 0.001; Fig. 5). The largest component was the costs for caregiver informal care, which represented over 50% of costs for each AD dementia severity group.

As with total societal costs, there was a significant increase in caregiver informal care costs and patient social care costs associated with increases in AD dementia severity (*p* < 0.001), but patient healthcare costs were not significantly increased (*p* = 0.624). The results of the sensitivity analyses confirmed these base case findings (data not shown).

## Discussion

This analysis from the GERAS observational study provides new information on clinical outcomes and societal costs associated with AD dementia in community-dwelling patients in the UK using prospective data. Deterioration in mean MMSE score over 18 months occurred at a similar rate in the mild and moderate AD dementia severity groups, with both showing a mean decrease of around 3.5 points over 18 months, and was greatest in the moderately severe/severe group (4.7 point decrease), although differences in MMSE score changes between the groups were not statistically significant. The probability of progression to institutionalisation or death increased in line with increases in baseline AD dementia severity.

Historically, the deterioration in MMSE score over time has been described as non-linear [24], as supported by a recently published, 18-month, randomised clinical trial in mild and moderate AD dementia [21]. In that study, MMSE scores in the mild and moderate groups receiving placebo decreased by an average of 2.4 and 5.8 points, respectively, over the 18-month period [21], and a similar decline in cognitive function was also seen in the same timeframe in a further study [22]. The results in our observational study did not demonstrate this trend. This disparity could be attributed to differences in study methodology, as randomised clinical trials are protocol-driven and patients are encouraged to stay within the trial; the greater patient retention rate observed in randomised clinical trials may mean that more information on patients’ MMSE scores over time is available. This difference in retention rates will be exaggerated by the inclusion of patients with more severe AD dementia within the GERAS study, where dementia severity was related to study retention. It is also known that the baseline demographics are different between the populations in these two types of studies.

In our analysis, 8.2% of patients with mild AD dementia at baseline had an MMSE score >26 at 18 months, while 48.3% of patients demonstrated a decline in MMSE score, with 11.6% transitioning from the mild to the moderately severe/severe group. Although variability between individual patients is often observed [25], some of these patients may not have had Alzheimer’s disease.

Our study demonstrated that patients with more severe dementia had a higher rate of institutionalisation and were more likely to die than those with less severe AD dementia. This is not surprising, and is consistent with the findings of a study conducted in 779 dementia patients recruited at nine memory centres, which demonstrated that a shorter time to institutionalisation was associated with lower cognitive ability, lower functional ability and more neuropsychiatric symptoms at baseline [26]. The greater the degree of deterioration in cognitive ability noted during the first 3 months of this study, the shorter the observed time to institutionalisation [26].

Consistent with baseline results [9], total societal costs increased with increasing baseline AD dementia severity, and caregiver informal care costs accounted for the highest proportion of the component costs in GERAS at all levels of severity. This latter finding potentially represents a large cost saving in terms of formal healthcare costs. A UK survey of caregivers of people with dementia found that many caregivers do not receive the support they need from health and care services, with key services such as home support for personal care, day care and provision of respite/time off from caring being either not offered or funded privately [27]. Carers should be provided with comprehensive support, including assistance with day-to-day caring, emotional support and regular, planned access to respite to support their own quality of life [4]. This is especially true given previous publications demonstrating an increase in caregiver burden with severity of AD dementia [28, 29].

The costs of Alzheimer’s disease to society observed in our study are substantial and were estimated to be approximately £26,000 for mild severity, £31,000 for moderate severity and £44,000 for the moderately severe/severe group per patient over an 18-month period. This translates to annual costs per patient of approximately £17,000 per year for mild, £21,000 for moderate and £29,000 for moderate/severe severity groups. These are comparable to the community figures calculated from the Alzheimer’s Society which have been rounded to annual rates per patient of £26,000 for mild, £43,000 for moderate and £55,000 for severe cases supporting the robustness of the data despite some methodological differences [1].

### Study strengths and limitations

Strengths of this study include that it was a large, prospective, observational study with a well-defined patient cohort, which collected longitudinal data over an 18-month period and assessed patients within the community across a range of disease severities. This study design would be expected to generate more reliable estimates of clinical outcomes, resource use and costs than either small cross-sectional studies [4, 30, 31] or retrospective database analyses, which may be susceptible to bias in data selection [32, 33].

The study also has limitations. Given the recruitment of patients principally from secondary care sites, it is possible that the sample is not truly representative of the overall AD dementia patient population. In addition, caregivers and participants were required to be sufficiently fit to participate in the study, which may represent a selection bias across all severities. It can also be challenging to make comparisons between studies due to different cost estimation methods, making analysis and interpretation of cost and resource data more difficult. Moreover, the 18-month duration of this study is still relatively short for accurately determining correlations between AD dementia severity and rate of cognitive decline or the probability of institutionalisation or death.

## Conclusions

This observational study demonstrated a rate of deterioration in cognition of at least 3.5 points on the MMSE scale over the 18-month study period across all patient groups stratified according to baseline AD dementia severity. Increasing severity of disease was associated with increased patient institutionalisation and death, together with increased total societal costs. Our results show that even when managed within the community, AD dementia is a costly disease, with costs associated with informal caregivers representing the main contributing factor to societal costs. Given the greater costs associated with increasing severity of disease, any treatment that could help slow disease progression may have a significant impact on the costs of community-based care for patients with AD dementia.

## Abbreviations

AD: Alzheimer’s disease
ADL: Activities of daily living
ANOVA: Analysis of variance
CI: Confidence interval
MMSE: Mini-mental state examination
MS/S: Moderately severe/severe
NICE: National Institute for Health and Care Excellence
RUD: Resource utilization in dementia
SIGN: Scottish Intercollegiate Guidelines Network
